# Relationship between “a body shape index (ABSI)” and body composition in obese patients with type 2 diabetes

**DOI:** 10.1186/s13098-018-0323-8

**Published:** 2018-03-20

**Authors:** Fernando Gomez-Peralta, Cristina Abreu, Margarita Cruz-Bravo, Elvira Alcarria, Gala Gutierrez-Buey, Nir Y. Krakauer, Jesse C. Krakauer

**Affiliations:** 1 Segovia General Hospital, C/Miguel Servet s/n, 40002 Segovia, Spain; 2 Department of Civil Engineering, The City College of New York, New York, NY USA; 3 Metro Detroit Diabetes and Endocrinology, Southfield, MI USA

**Keywords:** A body shape index, Sarcopenic obesity, Type 2 diabetes, Obesity, Body composition

## Abstract

**Background:**

Obesity is known to be related to the development of type 2 diabetes mellitus (T2D). The most commonly used anthropometric indicator (body mass index [BMI]) presents several limitations such as the lack of possibility to distinguish adipose tissue distribution. Thus, this study examines the suitability of a body shape index (ABSI) for prediction of body composition and sarcopenic obesity in obese or overweight T2D subjects.

**Methods:**

Cross-sectional study in 199 overweight/obese T2D adults. Anthropometric (BMI, ABSI) and body composition (fat mass [FM], fat-free mass [FFM], fat mass index [FMI] and fat-free mass index, and the ratio FM/FFM as an index of sarcopenic obesity) data was collected, as well as metabolic parameters (glycated haemoglobin [HbA_1c_], mean blood glucose, fasting plasma glucose [FPG], high-density-lipoprotein cholesterol [HDL], low-density-lipoprotein cholesterol, total cholesterol, and triglycerides [TG] levels; the ratio TG/HDL was also calculated as a surrogate marker for insulin resistance).

**Results:**

ABSI was significantly associated with age and waist circumference. It showed a statistically significant correlation with BMI exclusively in women. Regarding body composition, in men, ABSI was associated with FM (%), while in women it was associated with both FM and FFM. Both males and females groups with high ABSI scores were significantly older (men: 59.3 ± 10.8 vs 54.6 ± 10.1, p ≤ 0.05; women: 65.1 ± 9.8 vs 58.1 ± 13.3, p ≤ 0.005) and showed lower FFM values (men: 62.3 ± 9.0 vs 66.2 ± 9.3, p ≤ 0.05; women: 48.7 ± 5.6 vs 54.5 ± 8.9, p ≤ 0.001) compared with low-ABSI groups. Multiple linear regression revealed that ABSI independently predict FMI and the FM/FFM ratio in women. Sarcopenic obesity was identified in 70 (36.5%) individuals according to the FM/FFM ratio. The AUROC of ABSI was 63.1% (95% CI 54.6–71.6%; p = 0.003) and an ABSI value of 0.083 m^11/6^ kg^−2/3^ was the optimal threshold in discriminating patients with sarcopenic obesity (sensitivity: 48%, specificity: 73%). Moreover, a significant association between ABSI and FPG was found in men.

**Conclusions:**

ABSI could be useful to identify visceral and sarcopenic obesity in overweight/obese adults with T2D, adding some relevant clinical information to traditional anthropometric measures.

**Electronic supplementary material:**

The online version of this article (10.1186/s13098-018-0323-8) contains supplementary material, which is available to authorized users.

## Background

According to the global trends, the prevalence of diabetes mellitus is expected to increase considerably [1, 2]. Obesity, and especially visceral obesity, is known to be related to the development of type 2 diabetes (T2D), and cardiovascular diseases (CVD) [3, 4], through the release of fatty acids and inflammatory cytokines into the portal bloodstream [5].

Some anthropometric measurements considered surrogates for visceral obesity have long been used in medical settings for obesity-associated health risk evaluation. A commonly used measure is the body mass index (BMI). However, it does not serve to distinguish between muscle and fat accumulation and gives no indication of body shape [6]. An increase in BMI could be attributed to an increase in either fat mass (FM), fat-free mass (FFM) or both, limiting the utility of BMI to estimate adiposity [7, 8].

Waist circumference (WC) is widely seen as a measure of central adiposity. Its association with insulin resistance has been reported to be better than that of BMI [9–11]. Related indexes, such as waist-height ratio (WHtR), waist-hip ratio (WHR), and roundness index have been extensively studied. However, WC and WC-derived measures (i.e. WHtR) are highly correlated with BMI [12], limiting, their utility beyond BMI [13]. To overcome the limitations of the existing anthropometric measures to efficiently estimate both visceral abdominal and general adiposities and predict mortality.

Krakauer et al. [14] developed a new composite anthropometric measure. A body shape index (ABSI), based on normalizing WC to BMI and height. The advantage of ABSI is that it combines information from WC, height and weight. A high ABSI indicates that WC is higher than expected for a given height and weight, and corresponds to a more central concentration of body mass. ABSI predicts mortality independently from BMI [15, 16]. Of note, the DECODE study group found a positive linear relationship of all-cause CVD mortality with ABSI, whereas BMI, WC, and WHR showed J-shaped associations [17].

Additionally, ageing is usually associated with an increase in FM coexisting with a decrease in FFM, leading to sarcopenia [18]. Sarcopenia is characterized by degenerative loss of skeletal muscle mass and strength and correlates with physical disability [19]. In the case of sarcopenic obesity [20], ABSI may be useful, in addition to the traditional anthropometric measures.

The association of ABSI and body composition measurements has been reported in a geriatric study [21]. Overweight/obese subjects with no significant clinically comorbidities [22], and Japanese subjects with T2D [23] have also been studied. The methodology and the results differed between studies, but overall, they pointed out a positive relationship between ABSI and visceral and sarcopenic obesity.

Here we assess ABSI in an observational study of Caucasian obese or overweight subjects with T2D. Our findings suggest that ABSI is associated with body composition and sarcopenic obesity.

## Methods

### Design and study subjects

This cross-sectional observational study was carried out at the Hospital General de Segovia (Segovia, Spain) in accordance with the Declaration of Helsinki, including all amendments, and was approved by the corresponding Independent Research Ethics Committee. All participants provided written informed consent to use their data.

The study population comprised consecutive Caucasian overweight/obese (BMI ≥ 25 kg/m^2^) adult subjects with T2D. A glomerular filtration rate over 60 ml per minute per 1.73 m^2^ of body surface area (modification of diet in renal disease [MDRD] formula [24]) and normal creatinine levels were used to exclude clinically relevant renal impairment. Additional criteria for inclusion were not taking pioglitazone, GLP-1 receptor agonists or SGLT2 inhibitors, and performance of the studies required for this analysis.

### Measurements

#### Anthropometric parameters

Height (m) and weight (kg) were measured according to standard methods, and the BMI was then calculated as weight/height squared (kg/m^2^). Waist circumference (WC) was measured at the uppermost border of the iliac crests in standing positions with a non-stretchable tape (cm). ABSI was calculated as WC/(BMI^2/3^ * height^1/2^), expressed in m^11/6^ kg^−2/3^. Men and women were each divided into two groups by using the median of individual ABSI measurements as the threshold value. Subjects with ABSI lower than the median values were assigned to the “lower-ABSI” group and subjects with greater ABSI than the median value were assigned to the “higher-ABSI” group.

#### Body composition measurements

Fat mass (FM) and fat-free mass (FFM) were clinically determined by bioelectrical impedance analysis and calculated through the software supplied by the manufacturers and expressed in kg [25]. These measurements were performed in the morning, after an overnight fast. Subjects were asked to refrain from a strenuous exercise from the night before and to void the bladder before the examination. Fat mass index (FMI) and fat-free mass index (FFMI) were then calculated as FM (kg) and FFM (kg) divided by the squared height in meters (m^2^), respectively. The ratio between FM and FFM (FM/FFM) was calculated as an index of sarcopenic obesity. As per Prado et al. [26] we used the following cut-off values for FM/FFM ratio: < 0.40 for metabolic healthy obese individuals in whom the increase in FM relatively low compared to FFM; FM/FFM ratios between 0.40 and 0.80 for obese individuals; and FM/FFM > 0.80 for sarcopenic obese subjects, in whom FM predominates FFM.

#### Metabolic parameters

The collection of metabolic parameters included glycated haemoglobin (HbA1c), mean blood glucose (MBG), fasting plasma glucose (FPG), high-density lipoprotein cholesterol (HDL), low-density lipoprotein cholesterol (LDL), total cholesterol (TC), and triglycerides (TG) levels. The ratio between TG and HDL (TG/HDL) was also calculated as a surrogate marker for insulin resistance, as is suggested by Cordero et al. [19].

### Statistical considerations

To control for the well-known sexual dimorphism in body composition, all analyses were stratified by gender. Values were expressed as mean ± standard deviation (SD) for quantitative variables and as number and percentages for categorical variables. The Student’s t test or the Mann–Whitney U test for quantitative variables and the Fisher’s exact test or the Chi square test for qualitative variables were used to compare mean values between groups. Correlation analyses between anthropometric measures and body composition and metabolic parameters were evaluated with Pearson’s/Spearman correlation analysis. To determine if ABSI and BMI were independently associated with FM, FFM, FMI, FFMI, FM/FFM ratio, and TG/HDL index, linear regression analyses were performed. Correlations between anthropometric parameters and body composition measurements were adjusted for age, due to the documented relationship between the ageing process and changes in body composition, often without concomitant changes in body weight or BMI [20]. The area under the ROC curve (AUROC) was determined to compare the ability of ABSI and BMI to discriminate between patients with and without sarcopenic obesity. The significance level was set at p < 0.05 and all statistical analyses were carried out with the statistical package for the Social Sciences (SPSS) v.22.0 (SPSS, Inc., Chicago, IL).

## Results

Between March 2012 and April 2016, we identified 203 subjects who met the initial selection criteria; of these, four subjects were excluded due to screening failures (n = 2) and incomplete data (n = 2). Thus, the evaluable population comprised 199 subjects (100 women and 99 men), whose characteristics are shown in Table 1.

**Table 1:** Baseline characteristics

	Men n = 99	Women n = 100	*p* value
Age (years)	57.0 ± 10.7	61.5 ± 12.2	0.004
Duration of type 2 diabetes (years)	11.0 ± 6.9	12.9 ± 8.3	0.247
Height (cm)	167.9 ± 7.2	154.8 ± 7.0	< 0.001
Weight (kg)	102.7 ± 20.0	95.1 ± 19.3	0.007
BMI (kg/m^2^)	36.4 ± 6.5	39.7 ± 6.9	< 0.001
WC (cm)	118.3 ± 17.0	117.8 ± 14.9	0.791
ABSI (m^11/6^ kg^−2/3^)	0.0827 ± 0.0081	0.0810 ± 0.0072	0.001
Body fat (%)	36.7 ± 8.9	45.3 ± 5.2	< 0.001
FM (kg)	37.5 ± 15.5	44.0 ± 12.7	< 0.001
FFM (kg)	64.5 ± 9.4	51.4 ± 7.9	< 0.001
FMI	13.3 ± 5.5	18.3 ± 4.8	< 0.001
FFMI	22.8 ± 2.6	21.5 ± 2.9	< 0.001
FM/FFM ratio	0.59 ± 0.26	0.85 ± 0.16	< 0.001
< 0.4, n (%)	11 (11.5)	0 (0.0)	< 0.001
≥ 0.4 and ≤ 0.8, n (%)	76 (79.2)	35 (36.5)	
> 0.8, n (%)	9 (9.4)	61 (63.5)	
HbA1c (%)	8.4 ± 1.3	8.3 ± 1.7	0.109
MBG (mg/dL)	194.8 ± 38.5	190.5 ± 48.9	0.033
FPG (mg/dL)	182.0 ± 50.2	172.1 ± 73.0	0.109
HDL (mg/dL)	38.8 ± 11.7	44.8 ± 8.9	< 0.001
LDL (mg/dL)	99.8 ± 30.2	112.6 ± 40.6	0.060
TG (mg/dl)	202.7 ± 225.1	199.9 ± 182.6	0.246
TC (mg/dL)	176.1 ± 35.0	194.7 ± 57.7	0.003
TG/HDL	6.3 ± 9.5	4.7 ± 5.1	0.440
≤ 1.8, n (%)	7 (9.5)	7 (10.1)	> 0.999
> 1.8, n (%)	67 (90.5)	62 (89.9)	
Creatinine (mg/dL)	1.0 ± 0.2	0.8 ± 0.2	< 0.001
Glomerular filtration rate (mL/min/1.73 m^2^)^a^	91.5 ± 20.8	79.8 ± 18.9	< 0.001

Data are expressed as mean ± standard deviation, unless otherwise indicated

*ABSI* A body shape index, *BMI* body mass index, *FM* fat mass, *FFM* free-fat mass, *FMI* fat mass index, *FFMI* free-fat mass index, *FM/FFM* ratio fat mass-free-fat mass, *FPG* fasting plasma glucose, *HbA1c* haemoglobin A1c, *HDL* high-density lipoprotein cholesterol, *LDL* low-density lipoprotein cholesterol, *MBG* mean blood glucose, *TC* total cholesterol, *TG* Triglycerides, *WC* Waist circumference

^a^MDRD formula [25]

According to the FM/FFM ratio, 70 (36.5%) subjects had sarcopenic obesity, as defined by an FM/FFM ratio > 0.8. Only 9 of the male subjects showed sarcopenic obesity.

Pearson correlations between different variables are shown in Table 2 either adjusted or raw values. In both sexes, WC positively correlated with all body composition measurements. ABSI was significantly associated with age (men, r = 0.438, p = 0.000; women, r = 0.220, p = 0.029) and WC (men, r = 0.577, p = 0.000; women, r = 0.417, p = 0.000), and achieved a significant association with BMI only in women (r = -0.301, p = 0.003). Correlations between BMI and the body composition parameters were statistically significant for both men and women across all measures. Correlations between ABSI and body composition measurements in men were not significant except for the percentage of FM (r = − 0.302, p = 0.003), while in women ABSI significantly correlated with FM (r = − 0.236, p = 0.022) and FFM (r = − 0.270, p = 0.008). Overall, correlations between BMI and ABSI with metabolic parameters were not significant for either men or women (data not tabulated), except for the significant association found between ABSI and FPG in men (r = − 0.379, p = 0.000), and between BMI and TG in women (r = − 0.224, p = 0.043).

**Table 2:** Pearson’s rank correlation for body mass compositions measurements in men and women

	WC	% FM	BMI	Age	ABSI	FM	FFM	FMI	FFMI	FM/FFM
Men										
WC	1	0.260^f^	0.693^a^	0.136	0.577^a^	0.833^a^	0.376^a^	0.827^a^	0.499^a^	0.668^a^
% FM	0.322^g^	1	0.697^a^	− 0.313^g^	− 0.395^a^	0.892^a^	− 0.106	0.916^a^	− 0.069	0.932^a^
BMI	0.730^a^	0.691^a^	1	− 0.156	− 0.164	0.885^a^	0.389^a^	0.908^a^	0.553^a^	0.736^a^
Age	–	–	–	1	0.438^a^	− 0.225^h^	− 0.153	− 0.162	0.071	− 0.179
ABSI	0.580^a^	− 0.302^d^	− 0.107	–	1	− 0.124	− 0.164	− 0.092	− 0.023	− 0.061
FM	0.895^a^	0.888^a^	0.883^a^	–	− 0.030	1	0.211^i^	0.975^a^	0.175	0.923^a^
FFM	0.406^a^	− 0.164	0.374^a^	–	− 0.110	0.184	1	0.081	0.794^a^	-0.158
FMI	0.869^a^	0.924^a^	0.906^a^	–	− 0.023	0.976^a^	0.058	1	0.173	0.945^a^
FFMI	0.495^a^	− 0.049	0.573^a^	–	− 0.060	0.196	0.816^a^	0.188	1	− 0.135
FM/FFM	0.711^a^	0.938^a^	0.729^a^	–	0.020	0.921^a^	− 0.190	0.944^a^	− 0.124	1
Women										
WC	1	0.548^a^	0.722^a^	− 0.017	0.417^a^	0.827^a^	0.571^a^	0.849^a^	0.556^a^	0.696^a^
% FM	0.551^a^	1	0.631^a^	− 0.123	− 0.115	0.812^a^	0.323^b^	0.822^a^	0.225^h^	0.945^a^
BMI	0.723^a^	0.628^a^	1	− 0.087	− 0.301^d^	0.856^a^	0.742^a^	0.907^a^	0.794^a^	0.680^a^
Age	–	–	–	1	0.220^k^	− 0.236 ^l^	− 0.280^m^	− 0.110	− 0.029	− 0.110
ABSI	0.431^a^	− 0.091	− 0.291^r^	–	1	− 0.236^n^	− 0.270^j^	− 0.182	− 0.196	− 0.097
FM	0.847^a^	0.812^a^	0.863^a^	–	− 0.194	1	0.764^a^	0.944^a^	0.582^a^	0.856^a^
FFM	0.590^a^	0.303^d^	0.750^a^	–	− 0.222^s^	0.748^a^	1	0.657^a^	0.794^a^	0.335^b^
FMI	0.852^a^	0.820^a^	0.906^a^	–	− 0.163	0.950^a^	0.656^a^	1	0.684^a^	0.869^a^
FFMI	0.556^a^	0.224^q^	0.795^a^	–	− 0.194	0.593^a^	0.819^a^	0.685^a^	1	0.243^o^
FM/FFM	0.699^a^	0.944^a^	0.677^a^	–	− 0.075	0.859^a^	0.318^g^	0.867^a^	0.241^t^	1

The upper-right triangle of the table shows correlations of the raw values, while the lower-left triangle shows correlations adjusted for age

ap = 0.000; bp = 0.001; cp = 0.016; dp = 0.003; ep = 0.010; fp = 0.011; gp = 0.002; hp = 0.028; ip = 0.039; jp = 0.008; kp = 0.029; lp = 0.021; mp = 0.005; np = 0.022; op = 0.017; pp = 0.007; qp = 0.030; rp = 0.004; sp = 0.031; tp = 0.041

*ABSI* A body shape index, *BMI* body mass index, *% FM* Percentage of fat mass, *FM* fat mass, *FMI* fat mass index, *FFM* Fat-free mass, *FFMI* Fat-free mass index, *FM/FFM* ratio between fat mass and fat-free mass, *WC* waist circumference

**Fig. 1:**
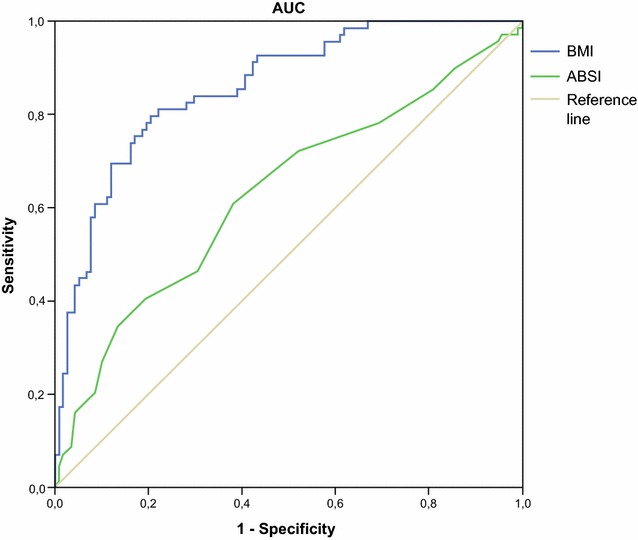
ROC curve for ABSI to discriminate between patients with and without sarcopenic obesity

The ROC curves of ABSI and BMI for predicting sarcopenia are shown in Fig. 1. Additionally, the AUROC of ABSI is shown in Fig. 1 and had a value of 63.1% (95% CI 54.6–71.6%; p = 0.003). We estimated a cut-off value of ABSI with potential clinical utility. In our analysis, the optimal threshold for the ABSI value in discriminating patients was 0.083 m^11/6^ kg^−2/3^ (sensitivity: 48%, specificity: 73%). For BMI, the cut-off value was 37,45 kg/m^2^, with sensitivity of 81% and specificity of 77%.

To selectively analyse the association between ABSI and anthropometric measures describing adiposity and sarcopenia independently from BMI, men and women were divided into two groups by using the median of individual ABSI measurements as the threshold value (0.0834 m^11/6^ kg^−2/3^). Higher-ABSI men had a mean WC significantly greater than the lower-ABSI men group (121.8 ± 13.6 vs 114.9 ± 19.4 cm, p ≤ 0.05). The higher-ABSI male and female groups were significantly older (age in years men: 59.3 ± 10.8 vs 54.6 ± 10.1, p ≤ 0.05; women: 65.1 ± 9.8 vs 58.1 ± 13.3, p ≤ 0.005). With higher-ABSI vs. lower-ABSI FFM was lower (men: 62.3 ± 9.0 vs 66.2 ± 9.3 kg, p ≤ 0.05; women: 48.7 ± 5.6 vs 54.5 ± 8.9, p ≤ 0.001), demonstrating the ability of ABSI to identify sarcopenia. Among women, BMI, FM, FMI, FFM, and FFMI exhibited significantly greater values in the lower-ABSI group than the higher-ABSI group (Table 3).

**Table 3:** Body and metabolic parameters in male and female subjects with lower- or higher-ABSI

	Men		Women	
	Lower-ABSI	Higher-ABSI	Lower-ABSI	Higher-ABSI
Age	54.6 ± 10.1	59.3 ± 10.8^a^	58.1 ± 13.3	65.1 ± 9.8^b^
BMI	37.1 ± 7.3	35.7 ± 5.9	42.0 ± 7.1	37.4 ± 6.1^c^
WC	114.9 ± 19.4	121.8 ± 13.6^a^	116.1 ± 16.5	119.5 ± 13.1
% of FM	37.8 ± 10.2	35.8 ± 7.5	45.9 ± 5.3	44.7 ± 5.2
FM	39.6 ± 17.8	35.5 ± 13.3	47.6 ± 13.3	41.0 ± 11.6^a^
FFM	66.2 ± 9.3	62.3 ± 9.0^a^	54.5 ± 8.9	48.7 ± 5.6^c^
FMI	13.9 ± 6.4	12.9 ± 4.6	19.6 ± 4.8	17.2 ± 4.6^a^
FFMI	22.9 ± 2.5	22.6 ± 2.8	22.5 ± 3.0	20.6 ± 2.4^c^
FM/FFM	0.60 ± 0.30	0.58 ± 0.24	0.87 ± 0.16	0.83 ± 0.16
HbA1c	8.3 ± 1.3	8.6 ± 1.4	8.1 ± 1.7	8.3 ± 1.6
HDL	37.9 ± 10.8	39.9 ± 12.7	43.2 ± 8.6	46.1 ± 8.9
LDL	93.4 ± 29.1	107.2 ± 30.9	106.0 ± 39.0	119.8 ± 42.3
TG	201.5 ± 200.8	202.2 ± 253.8	161.8 ± 79.5	244.3 ± 250.8
TC	170.4 ± 32.8	182.5 ± 36.7	179.4 ± 37.3	212.3 ± 71.6^b^
TG/HDL	6.1 ± 8.0	6.6 ± 11.0	4.1 ± 2.4	5.4 ± 7.0

Data are expressed as mean ± standard deviation

*BMI* body mass index, *% FM* Percentage of fat mass, *FM* fat mass, *FMI* fat mass index, *FFM* Fat-free mass, *FFMI* Fat-free mass index, *FM/FFM* ratio between fat mass and fat-free mass, *HDL* High-density lipoprotein cholesterol, *LDL* Low-density lipoprotein cholesterol, *TG* Triglycerides, *TC* Total cholesterol, *WC* waist circumference

^a^ p ≤ 0.05, ^b^ p ≤ 0.005, ^c^ p ≤ 0.001

Multiple linear regression analyses revealed that BMI independently predicted all body composition parameters both in men and women. ABSI, however, independently predicted FMI and FM/FFM ratio in women (Additional file 1: Table S1).

## Discussion

This study utilizes indexes defining body composition that are easily obtained in routine clinical practice. It assesses the relationship between the components of body composition and abdominal fat surrogate markers in a retrospective Spanish cohort overweight or obese adults with T2D.

In our series, we found that BMI was highly and positively correlated with all parameters of body composition (i.e. FM, FFM, FMI, etc.), in both males and females. The observed lack of correlation between ABSI and BMI in the general population was confirmed in the male cohort, while there was a modest correlation in the female cohort. This is in line with the recent study conducted by Hardy et al. [27] that aimed to determine the best anthropometric discriminators of T2D among white and black subjects in a large cohort. It was concluded that those anthropometric measures that included WC (such as WHR) were the strongest discriminators of T2D across race-gender groups, while BMI was a comparable discriminator to WC among males, but not in female subjects.

ABSI was positively correlated with WC both in males and females. A higher ABSI-value is an indication of a higher abdominal fat deposition [14] that leads to systemic inflammation [28], insulin resistance [29], and accompanies a systemic loss of skeletal muscle mass, as reported previously [30]. In our study, the association of ABSI with body composition parameters differed by gender: ABSI was negatively associated with the percentage of FM (p = 0.000) in the male cohort, while it was negatively associated with both FM (p = 0.022) and FFM (p = 0.008) in the female cohort. This discrepancy in the findings between men and women could be explained by the differences in body fat distribution [31]. In men, adipose tissue tends to be more centrally deposited, suggesting that WC would be sensitive to FM, whereas in women adipose tissue is mainly deposited in the lower body (gluteal-femoral), indicating that WC may be less sensitive to FM [32].

The higher-ABSI females and males showed significantly lower FFM than the lower-ABSI groups, with comparable BMI in men and lower BMI in women. These findings could support the hypothesis that abdominal fat deposition may be associated with a loss of skeletal muscle mass in some subjects, i.e. sarcopenic obesity. Obesity-related sarcopenia, is a syndrome of progressive and generalised loss of skeletal muscle mass and function, characterized by a higher FM in relation to FFM [26]. Our study indicates that ABSI may not be just a marker of visceral obesity, but may also represent an index of decreased muscle mass (i.e. sarcopenia) in T2D subjects, which is congruent with the positive correlation of ABSI and age generally reported and also found in our sample.

The AUROC analysis supports that ABSI showed ability for predicting the presence of sarcopenic obesity (the lower bound of the 95% CI of AUC is greater than 0.5; [33]). Even though the ability of BMI to discriminate between patients with and without sarcopenic obesity is higher than for ABSI, as indicated by the respective sensitivity and specificity values, ABSI could add relevant information about abdominal obesity, body composition and mortality risk. In fact, our proposed ABSI cut-off point diagnostic of sarcopenic obesity (0.083 m^11/6^ kg^−2/3^) exactly agreed with the finding of a previous study [14], where ABSI above the same threshold predicted a higher mortality relative hazard.

The data presented add to the emerging, growing body of literature regarding different aspects of ABSI. A recent longitudinal study conducted with Australian adults showed a positive association between ABSI and mortality, suggesting that this measure could be a useful predictor of mortality hazard in different populations [34]. In this context, further studies are needed to investigate whether ABSI could be used as an indicator of the effectiveness of lifestyle modifications in T2D subjects.

The authors recognise some limitations in the study that should be considered when interpreting the results. First of all, this is an observational study with a cross-sectional design; therefore, there is no patient follow-up, which would be of importance to elucidate the correlation between body composition and ABSI; besides, we cannot rule out that some degree of bias may have been introduced. Secondly, as this study was performed at a single centre, findings in other centres may be different and, certainly, further confirmation is needed. Nonetheless, all these findings should guide further prospective studies with some broader samples [34].

In conclusion, ABSI identifies visceral obesity and sarcopenia in overweight or obese subjects with T2D, adding some relevant clinical information to traditional anthropometric measures.

## Additional file

**Additional file 1: Table S1.** Linear regression analysis of ABSI and BMI with body composition measurements.

## Abbreviations

ABSI: a body shape index
BMI: body mass index
CVD: cardiovascular disease
FFM: fat-free mass
FM: fat mass
FPG: fasting plasma glucose
HbA1c: glycated haemoglobin
HDL: high-density lipoprotein
LDL: low-density lipoprotein
MBG: mean blood glucose
SD: standard deviation
T2D: type 2 diabetes
TC: total cholesterol
TG: triglycerides
WC: waist circumference
WHR: waist-hip ratio
WHtR: waist-height ratio
