# Supplementary material for: Relationship between “a body shape index (ABSI)” and body composition in obese patients with type 2 diabetes
Source: Diabetol Metab Syndr. 2018 Mar 20;10:21. doi: 10.1186/s13098-018-0323-8 (PMC5859756; doi:10.1186/s13098-018-0323-8)
Supplement: Supplementary file 1 — Additional file 1: Table S1. Linear regression analysis of ABSI and BMI with body composition measurements. [file 13098_2018_323_MOESM1_ESM.docx]

# SUPPLEMENTARY MATERIAL

**Table 1** Linear regression analysis of ABSI and BMI with body composition measurements

|  | **Men** | **Women** |
| --- | --- | --- |
|  | **β coefficients (95% CI)** | **β coefficients (95% CI)** |
| FM (dependent variable) |  |  |
| ABSI | -152.57 (-557.54 – 252.39) | 186.97 (-87.73 – 461.68) |
| BMI | 2.09 (1.86 – 2.32)^a^ | 1.61 (1.40 – 1.82)^a^ |
| FFM (dependent variable) |  |  |
| ABSI | -185.15 (-466.21 – 95.90) | -53.83 (-210.69 – 103.03) |
| BMI | 0.54 (0.28 – 0.81)^a^ | 0.83 (0.67 – 0.99)^a^ |
| FMI (dependent variable) |  |  |
| ABSI | -1.58 (-131.16 – 128.00) | 143.99 (64.40 – 223.57)^b^ |
| BMI | 0.76 (0.67 – 0.84)^a^ | 0.66 (0.60 – 0.72)^a^ |
| FFMI (dependent variable) |  |  |
| ABSI | 14.04 (-58.48 – 86.56) | 18.83 (-33.63 – 71.28) |
| BMI | 0.22 (0.15 – 0.29)^a^ | 0.33 (0.28 – 0.39)^a^ |
| FM/FFM (dependent variable) |  |  |
| ABSI | 0.93 (-9.11 – 10.97) | 5.09 (0.11 – 10.07)^c^ |
| BMI | 0.03 (0.02 – 0.04)^a^ | 0.02 (0.01 – 0.02)^a^ |
| TG/HDL (dependent variable) |  |  |
| ABSI | -81.79 (-704.12 – 540.54) | 99.05 (-148.26 – 346.36) |
| BMI | 0.05 (-0.27 – 0.37) | -0.08 (-0.28 – 0.12) |
| ^a^p= 0.000; ^b^p= 0.001; ^c^p≤0.05 | | |
| *ABSI* A body shape index, *BMI* body mass index, *CI* confidence interval, *FM* fat mass, *FMI* fat mass index, *FFM* Fat-free mass, *FFMI* Fat-free mass index, *FM/FFM* ratio between fat mass and fat-free mass, *TG/HDL* ratio between triglycerides and high-density cholesterol. | | |
